# Consistently Low Levels of Osteocalcin From Late Pregnancy to Postpartum Are Related to Postpartum Abnormal Glucose Metabolism in GDM Patients

**DOI:** 10.3389/fendo.2022.803624

**Published:** 2022-03-07

**Authors:** Yujia Gong, Na Li, Mengyu Lai, Fang Fang, Jiaying Yang, Mei Kang, Tingting Shen, Yongde Peng, Yufan Wang

**Affiliations:** ^1^ Department of Endocrinology and Metabolism, Shanghai General Hospital, Shanghai Jiao Tong University School of Medicine, Shanghai, China; ^2^ Clinical Research Center, Shanghai General Hospital, Shanghai Jiao Tong University School of Medicine, Shanghai, China

**Keywords:** osteocalcin, gestational diabetes mellitus, abnormal glucose metabolism, risk factors, postpartum glucose metabolism

## Abstract

**Objective:**

Increasing evidence suggests that osteocalcin (OC), a marker of bone formation, plays an important role in glucose homoeostasis. Few studies have investigated the relationship between OC levels in gestational diabetes mellitus (GDM) patients and their postpartum glucose metabolism. This study evaluated the relationship between OC levels in late pregnancy, their longitudinal changes, and postpartum glucose metabolism among GDM patients.

**Measures:**

Serum OC was measured in late pregnancy and the postpartum period for 721 GDM patients. All patients underwent a 75-g oral glucose tolerance test (OGTT) at 6–8 weeks postpartum. According to postpartum OGTT outcomes, patients were categorized into abnormal glucose metabolism (AGM) (n=255) and normal glucose tolerance (NGT) groups (n=466). Glucose metabolism-related indices were measured and calculated. Logistic regression analysis and linear mixed-effects model were used to assess the association between OC and postpartum AGM.

**Results:**

In late pregnancy, OC levels were lower in the AGM group than in the NGT group (13.93 ± 6.90 vs 15.33 ± 7.63 ng/ml, *P*=0.015**).** After delivery, OC levels increased in both groups. However, OC levels remained lower in the AGM group than in the NGT group (23.48 ± 7.84 vs 25.65 ± 8.37 ng/ml, *P*=0.001). Higher OC levels in late pregnancy were associated with decreased risk of progressing to postpartum AGM (OR:0.96, 95%CI:0.94–0.99). Linear mixed-effects analysis showed that postpartum AGM patients exhibited consistently lower OC levels than NGT group from late pregnancy to the postpartum period after adjustment for cofactors (β=-1.70, 95% CI: -2.78– -0.62).

**Conclusions:**

In GDM patients, consistently low levels of OC from late pregnancy to postpartum were associated with increased postpartum AGM risk. The increase in serum OC may act as a protective factor to curb the progression of AGM at postpartum for GDM patients.

## Introduction

Recently, bone has been identified as an endocrine organ involved in energy metabolism through the secretion of specific hormones (1, 2). Osteocalcin (OC), a small noncollagenous protein of 49 amino acids that is exclusively secreted by osteoblasts, participates in bone remodeling and calcium homeostasis. OC has three γ-carboxyglutamic acid residues in the 17, 21 and 24 positions of its peptide chain, which undergoes a posttranslational modification at the glutamate residue to attain a higher affinity for hydroxyapatite to integrate into the bone extracellular matrix (1, 3, 4). However, the undercarboxylated form (ucOC), as a bioactivator released into the circulation, may serve as a modulator of energy metabolism. Since ucOC levels are difficult to measure, most studies have focused on total OC (5–7).

Accumulating evidence shows that OC is vital in the cross-talk between bone remodeling and energy metabolism. Extensive animal studies have shown that OC stimulates insulin secretion directly by exerting an effect on pancreatic β-cell and indirectly *via* the secretion of glucagon-like peptide (GLP-1) by enteroendocrine L cells leading to improved insulin sensitivity (8, 9). In contrast, osteocalcin-deficient mice displayed decreased β-cell proliferation, glucose intolerance, and insulin resistance (10). To date, almost human studies have supported the findings of animal studies. In humans, serum OC was reported to be decreased in patients with type 2 diabetes compared to the levels in nondiabetic controls; inversely associated with blood glucose levels, HbA1c, BMI and insulin resistance; and positively associated with insulin secretion and insulin sensitivity (7, 11, 12).

Gestational diabetes mellitus (GDM), defined as hyperglycemia first recognized during pregnancy, is one of the most common metabolic complications in pregnancy (13, 14). Although glucose intolerance in many GDM patients usually reverts to normal after delivery, these patients and their offspring face an increased lifetime risk of developing type 2 diabetes mellitus (T2DM) and cardiovascular diseases (CVD) in the future (15–17). As T2DM can be prevented or delayed by intensive lifestyle or metformin intervention (18, 19), it is suggested that GDM patients should be routinely screened, which is beneficial for early intervention (20).

A few studies have previously assessed the contribution of serum OC in this context. Higher OC concentrations in GDM patients than in euglycemic pregnant women and a positive association between OC and insulin resistance parameters during pregnancy had been reported (21–23), and these findings are in contrast to what has been observed in the context of diabetes. A possible explanation for the opposite results in GDM could be an early adaption to impaired glucose tolerance.

Although a few studies have explored the effects of OC on glucose metabolism in GDM patients, the role of OC levels in the postpartum glucose metabolism of GDM is unclear. Therefore, we studied 721 GDM patients and evaluated OC levels both in late pregnancy and postpartum. The associations of OC levels and their longitudinal trajectory changes with postpartum glucose metabolism of GDM were explored in our study.

## Materials and Methods

### Patient Population

This retrospective study was performed at the Department of Endocrinology and Metabolism of Shanghai General Hospital from December 2015 to December 2020. Pregnant women underwent a 75-g OGTT test at 24-28 weeks of gestation and the International Association of Diabetes and Pregnancy Study Groups (IADPSG) criteria was used for the diagnosis of GDM (24): fasting plasma glucose (FPG) value ≥5.1 mmol/L and/or 1-h postprandial glucose (1h-PG) value ≥10.0 mmol/L and/or 2-h postprandial glucose (2h-PG) value ≥8.5 mmol/L. After delivery, all individuals with GDM were invited to undergo a 75-g OGTT test again at 6–8 weeks postpartum. Subjects with a history of diabetes mellitus (DM) or impaired fasting glucose (IFG) and/or impaired glucose tolerance (IGT) before pregnancy or lack of OC either in late gestation or at postpartum were excluded. Finally, a total of 721 subjects were included in this study.

Subjects were categorized into two groups according to 75-g OGTT results at 6–8 weeks postpartum based on 1999 WHO criteria (25): 1. Abnormal glucose metabolism (AGM) group: IFG (6.1 mmol/L ≤ FPG <7.0 mmol/L and 2h-PG <7.8 mmol/L) or IGT (FPG < 7.0 mmol/L and 7.8 mmol/L ≤ 2h-PG <11.1 mmol/L) or DM (FPG ≥7.0 mmol/L or/and 2h-PG ≥11.1 mmol/L); 2. Normal glucose tolerance (NGT) group: FPG <6.1 mmol/L and 2h-PG <7.8 mmol/L.

This study was approved by the institutional ethics committee of Shanghai General Hospital.

### Study Protocol and Methods

Clinical data including age at present pregnancy, family history of diabetes, parity, pregestational body mass index (pre-BMI), BMI at 6–8 weeks postpartum, OC levels and other clinical indexes of glucose and lipid metabolism were recorded. BMI was calculated as the weight in kilograms divided by the square of the height in meters (kg/m^2^). Homeostasis model assessment was used to estimate insulin resistance (HOMA-IR) which was defined as [fasting insulin (μU/ml) * fasting glucose (mmol/l)]/22.5, and HOMA of β-cell (HOMA-β) index was used to assess β-cell function, which was calculated as [20*fasting insulin (μU/ml)]/[fasting glucose (mmol/l) – 3.5].

All blood samples were obtained in the morning after an overnight fast of 8–10 h. In our study, we used N-terminal mid-fragment of OC (N-MID OC), the largest proteolytic fragment with a relatively long half-life, to reflect serum OC levels (26). N-MID OC was measured using electrochemiluminescent immunoanalysis (Roche Cobas e601, Germany). HbA1c was measured with an autoanalyzer (Lifotronic H8, Japan). Serum insulin was measured using an automated chemiluminescence systems (Abbott i2000, United States). Serum glucose and lipid profiles including serum total cholesterol (TC), triglycerides (TGs), high-density lipoprotein cholesterol (HDL-C) and low-density lipoprotein cholesterol (LDL-C), were measured with an automatic biochemistry analyzer (Siemens ADVIA2400, Germany). During the study period, instruments or testing methodologies unchanged.

### Statistical Analysis

Data are presented as the mean ± standard deviation (SD) or median (interquartile range, 25–75%) for continuous variables and proportion for categorical variables, respectively. Normally distributed continuous variables were compared by Student’s t test, while nonnormally distributed continuous variables were analyzed by the Mann-Whitney U test. Categorical variables were analyzed byχ^2^ test. The log-transformed levels of HOMA-β were parameterized as a continuous variable. The Pearson correlation coefficients were calculated to assess the strength of the correlation of OC in late gestation and glucose related indicators, insulin resistance and β-cell function. Multivariate linear regression was performed to determine the associations between OC levels and insulin resistance, and β-cell function.

Multiple logistic regression models were used to calculate the odds ratio (OR) and 95% CIs for the risk of postpartum AGM for OC levels in late gestation. *A priori* selection of conventional postpartum AGM risk factors, including age, postpartum BMI, parity, family history of diabetes and HbA1c in late pregnancy, was assessed at study enrollment. A linear mixed-effects model was performed to compare the longitudinal trajectories of OC in late gestation and postpartum in individuals with different postpartum glucose status according to 75-g OGTT results by using restricted maximum likelihood estimation. The model included serum OC in late pregnancy and postpartum, groups of different postpartum OGTT outcomes and time in late pregnancy and at postpartum. OC levels were adjusted for maternal age, prepregnancy BMI, family history of diabetes, parity and HbA1c in late gestation *via* covariate adjustment (fixed effects in the mixed model).

All statistical analyses were performed using SPSS version 26 (IBM Corp., Armonk, NY), and a *P* value<0.05 was considered statistically significant.

## Results

The mean age in the cohort was 31.98 ± 4.4 years. All GDM patients received lifestyle modification and 174 (24%) women received additionally insulin therapy during pregnancy. According to the results of the postpartum OGTT, 255 individuals were diagnosed with AGM, of whom 221 had IFG and/or IGT and 34 had diabetes. The remaining 466 women had normal glucose tolerance.

The baseline characteristics of the GDM subjects stratified by the outcomes of postpartum 75-g OGTT were shown in **Table 1**. Compared with the NGT group, subjects in the AGM group were older and had higher BMI and HbA1c both before and after delivery. Meanwhile, postpartum FBG, 2h-PG, 2h-INS, TC, TGs and LDL-C were significantly higher in the AGM group. Indices of insulin resistance (HOMA-IR) indicated that postpartum AGM women were more insulin resistant than NGT women. The OC levels of the AGM group were lower both in late pregnancy (NGT vs AGM: 15.33 ± 7.63 ng/ml vs 13.93 ± 6.90 ng/ml, *P*=0.015) and postpartum (NGT vs AGM: 25.65 ± 8.37 ng/ml vs 23.48 ± 7.84 ng/ml, *P*=0.001) (**Table 1**).

**Table 1:** Characteristics and metabolic parameters of GDM women with different glucose outcomes according to the 75-g OGTT at 6–8 weeks postpartum.

	75-g OGTT results at 6–8 weeks postpartum		P value
	NGT (n=466)	AGM (n=255)	
Age (years)	31.62 ± 4.17	32.64 ± 4.74	0.004
Family history of diabetes	101 (21.7%)	52 (20.6%)	0.715
Primiparity	245 (53.0%)	128 (50.6%)	0.533
Pre-BMI (kg/m^2^)	22.50 ± 3.43	23.25 ± 3.56	0.006
Postpartum BMI (kg/m^2^)	23.72 ± 3.21	24.22 ± 3.32	0.046
HbA1c in late pregnancy (%)	5.45 ± 0.44	5.56 ± 0.51	0.005
OC level in late pregnancy (ng/ml)	15.33 ± 7.63	13.93 ± 6.90	0.015
Laboratory values at postpartum			
FBG (mmol/L)	4.91 ± 0.52	5.24 ± 0.85	<0.001
2h-PG (mmol/L)	6.33 ± 0.90	9.20 ± 1.52	<0.001
TCH (mmol/L)	5.30 ± 0.96	5.48 ± 0.95	0.017
TGs (mmol/L)	1.19 ± 0.78	1.37 ± 0.97	0.01
HDL-C (mmol/L)	1.49 ± 0.38	1.47 ± 0.34	0.535
LDL-C (mmol/L)	3.01 ± 0.81	3.15 ± 0.85	0.027
FINS (mU/L)	6.65 ± 4.30	7.33 ± 4.90	0.064
2h-INS (mU/L)	22.44 (15.47, 32.85)	37.96 (24.47, 56.64)	<0.001
HOMA-IR	1.47 ± 1.00	1.77 ± 1.35	0.002
HOMA-β	84.48 (57.47, 130.43)	78.29 (53.54, 115.03)	0.070
HbA1c (%)	5.36 ± 0.43	5.55 ± 0.52	<0.001
OC at postpartum (ng/ml)	25.65 ± 8.37	23.48 ± 7.84	0.001

Data are presented as the mean ± SD, median (interquartile range), or n (%) as appropriate.

OGTT, oral glucose tolerance test; NGT, normal glucose tolerance; AGM, abnormal glucose metabolism; Pre-BMI, body mass index before pregnancy; HbA1c, glycated haemoglobin; OC, osteocalcin; FBG, fasting blood glucose; 2h-PG, 2-h postprandial glucose; TCH, total cholesterol; TGs, triglycerides; HDL-C, high-density lipoprotein cholesterol; LDL-C, low-density lipoprotein cholesterol; FINS, fasting insulin; 2h-INS, 2-h postprandial insulin; HOMA-IR, homeostasis model assessment of insulin resistance; HOMA-β, homeostasis model assessment of beta-cell function.

**Figure 1:**
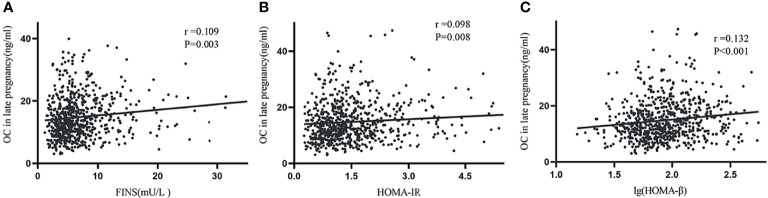
Simple correlations between late pregnancy OC and postpartum FINS, HOMA-IR, and lg (HOMA-β). Serum OC in late pregnancy was positively associated with FINS **(A)**, HOMA-IR **(B)** and lg (HOMA-β) **(C)**.

The correlation analysis showed that OC levels were positively associated with postpartum FINS (r=0.109, *P*=0.003), HOMA-IR (r=0.098, *P*=0.008) and lg (HOMA-β) (r=0.132, *P*<0.001) (**Figure 1**), but had no relationship with postpartum FBG and HbA1c (data not shown). In order to further explore the relationship between OC levels and insulin resistance and β-cell function, multivariate linear regression was used. We found that lg (HOMA-β) was positively associated with OC levels after adjusted for age, postpartum BMI, parity, family history of diabetes and HbA1c in late pregnancy (β=0.003, *P*=0.015), while HOMA-IR was not associated with OC after adjusted covariates above (β=0.002, *P*=0.783).

Logistic regression analysis revealed that the risk of developing AGM at postpartum was decreased by 3% after adjusting for age and parity (OR=0.97, 95%CI: 0.95-0.99). This association remained significant after further adjustment for postpartum BMI, family history of diabetes and HbA1c in late pregnancy (OR=0.96, 95%CI: 0.94-0.99) (**Table 2**).

**Table 2:** Logistic regression analysis showing the association between OC in late pregnancy and postpartum AGM.

Factors	OR	95%CI	P value
Age (years)	1.05	1.01-1.1	0.018
Postpartum BMI (kg/m^2^)	1.04	0.99-1.1	0.126
Family history of diabetes: no (reference)	0.89	0.6-1.33	0.573
Parity: 1 (reference)	0.80	0.55-1.16	0.243
OC level in late pregnancy (ng/ml)	0.96	0.94-0.99	0.004
HbA1c in late pregnancy (%)	1.61	1.11-2.34	0.012

BMI, body mass index; OC, osteocalcin; HbA1c, glycated haemoglobin.

**Figure 2:**
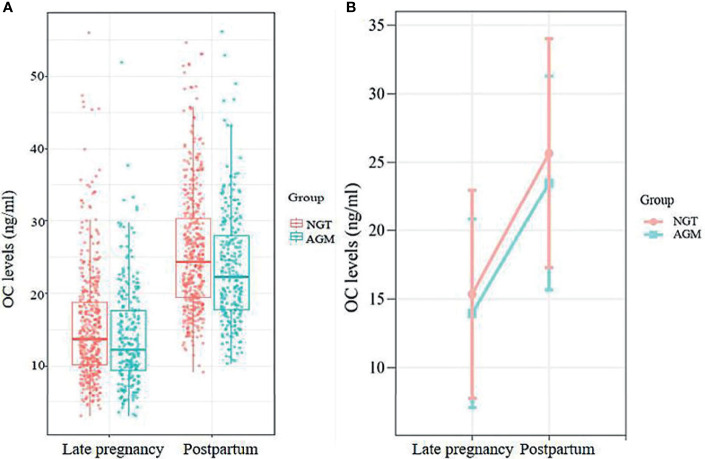
**(A)** The distributions of OC levels at late pregnancy and postpartum in the NGT and AGM group. **(B)** Longitudinal change in OC levels in postpartum AGM individuals (orange line) and NGT individuals (blue line), adjusted for maternal age, parity, family history of DM, pre-BMI, and HbA1c in late pregnancy.

OC levels increased significantly after delivery in both the NGT (from 15.33 ± 7.63 in late gestation to 25.65 ± 8.37 at postpartum, *P*<0.001) and AGM (from 13.93 ± 6.90 in late gestation to 23.49 ± 7.84 at postpartum, *P*<0.001) groups (**Figure 2**). Meanwhile, the linear mixed-effects model showed that the OC levels from late pregnancy to postpartum were consistently lower in AGM group than in NGT group, adjusted for parity, age, time points (late pregnancy and postpartum), pre-pregnancy BMI, HbA1c and family history of diabetes (β=-1.70, 95% CI: -2.78– -0.62) (**Supplementary Table 1**).

## Discussion

In the current study, we investigated the association between serum OC levels and the postpartum glucose metabolism of GDM. We found that low serum OC in late pregnancy was associated with increased risk of developing postpartum AGM. After delivery OC levels increased significantly in both groups, however, the OC levels were consistently low from late pregnancy to postpartum in the AGM group than in the NGT group. To the best of our knowledge, this is the first study exploring the relationship between longitudinal changes in OC and the postpartum glucose metabolism of GDM.

In animal and clinical investigations, OC, a traditional bone formation marker, has been found to participate in the regulation of glucose metabolism. Some evidence from animal studies suggested that higher OC concentrations are protective against diet-induced obesity and type 2 diabetes. Mice lacking the *Esp* gene, which encoded osteotesticular protein tyrosine phosphatase (OST-PTP), a receptor-like protein that inhibited the bioactivity of osteocalcin, exhibited hypoglycemia and were protected from glucose intolerance due to increases in pancreatic β-cell proliferation, insulin secretion and insulin sensitivity (10). In contrast, *Osteocalcin-* knockout mice had the opposite phenotypes, namely glucose intolerance and obesity (10). On the other hand, infusion *via* subcutaneous minipump or daily injections or oral administration of recombinant OC significantly improved glucose tolerance and insulin sensitivity in mice fed a normal diet, which was possibly attributed to an increase in both β-cell mass and insulin secretion (27–29). In addition, OC stimulates pancreatic β-cell proliferation in cultured human islets (30). In accordance with previous animal studies, two meta-analyses confirmed that lower OC levels were observed in patients with type 2 diabetes than in normal controls (7, 31) and acknowledged that OC was negatively associated with fasting plasma glucose levels, HbA1c, insulin resistance and body mass index (BMI) but positively correlated with improved glycemic control, weight loss and regular exercise (11, 12).

Pregnancy itself was an insulin-resistant physiological state, and by the end of pregnancy, insulin sensitivity decreased by roughly 50% (32). To maintain euglycemia, insulin secretion increased 3 to 3.5-fold to protect against insulin resistance (33). After delivery, women’s insulin sensitivity increased rapidly by 120% compared with that during late pregnancy. However, women with previous GDM remained in a state of chronic inflammation and insulin sensitivity did not significantly improve (34). In the long run, individuals with a history of GDM seemed to have an approximately 10 times higher risk of developing T2DM than those with NGT during pregnancy (35). An increasing number of studies have explored the role of OC in GDM previously, but many of them focused on the difference in OC levels between GDM patients and normal controls (21, 23). There were limited studies on the relationship between OC changes and postpartum glucose metabolism in GDM patients.

Winhofer et al. (21) found that OC levels increased in all women at 12 weeks postpartum, which was confirmed in Saucedo et al. research (36). However, fewer than 100 women with GDM underwent postpartum follow-up in their studies. In our study, we included more than 700 GDM patients. We found a 35.4% incidence of AGM; among the individuals with AGM, 13.3% were diagnosed with diabetes at 6–8 weeks postpartum. Consistent with the previous research by Saucedo et al. (36), our study showed that in the GDM group, subjects who progressed to AGM at postpartum had lower OC concentrations than individuals in the NGT group. Furthermore, we found that the OC levels from late pregnancy to postpartum were consistently lower in AGM group than in NGT group. Considering that OC can stimulate insulin secretion and has been shown to have a beneficial effect on glucose metabolism in animal and human studies, we speculated that the consistently lower levels in OC in the postpartum AGM group was an insufficient compensation for insulin resistance.

To test this hypothesis, we conducted simple correlations and found that serum OC was positively related with HOMA-β and the positive association was still robust after adjusting for age, postpartum BMI, parity, family history of diabetes and HbA1c in late pregnancy. Multivariate regression models further revealed that the risk of progressing to postpartum AGM decreased by 3.6% with per 1ng/ml increment of serum OC in late pregnancy (OR:0.964, 95%CI:0.940-0.988). Therefore, it is likely that in GDM patients, OC increases as a protective compensation mechanism to stimulate insulin secretion to cope with increased insulin demand and to further prevent developing of AGM.

There are several limitations in our study. First, we measured only the N-MID OC not ucOC, the bioactive form of OC, which is difficult to measure (37, 38). However, N-MID OC is the most stable form of OC in serum (26). Second, we observed the relationship between OC and postpartum AGM in only a short period. Therefore, it is necessary to conduct prospective and mechanistic studies in the future.

In conclusion, consistently low levels of osteocalcin from late pregnancy to postpartum in GDM patients were at high risk of postpartum AGM. Increasing serum OC levels may become a potential preventive indicator to curb the progression to postpartum IFG/IGT or even T2DM.

## Data Availability Statement

The raw data supporting the conclusions of this article will be made available by the authors, without undue reservation.

## Ethics Statement

The studies involving human participants were reviewed and approved by The institutional ethics committee of Shanghai General Hospital. The ethics committee waived the requirement of written informed consent for participation.

## Author Contributions

YG and NL conceived of the design of the study and drafted the manuscript. ML, FF, and JY contributed to the data collection. MK and TS participated in the data analysis. YW and YP critically reviewed the data and the manuscript. All authors have reviewed and approved the final manuscript, contributed to the design of the study and interpretation of the data.

## Funding

National Natural Science Foundation of China (No. 81870610 & No. 82170879), the Shanghai Science and Technology Commission Foundation (No. 21Y11904800), Clinical Research Plan of SHDC (No. SHDC2020CR3065B).

## Conflict of Interest

The authors declare that the research was conducted in the absence of any commercial or financial relationships that could be construed as a potential conflict of interest.

## Publisher’s Note

All claims expressed in this article are solely those of the authors and do not necessarily represent those of their affiliated organizations, or those of the publisher, the editors and the reviewers. Any product that may be evaluated in this article, or claim that may be made by its manufacturer, is not guaranteed or endorsed by the publisher.

## References

[1] Zoch ML Clemens TL Riddle RC. New Insights Into the Biology of Osteocalcin. Bone (2016) 82:42–9. doi: 10.1016/j.bone.2015.05.046 PMC4670816 26055108

[2] Al-Suhaimi EA Al-Jafary MA. Endocrine Roles of Vitamin K-Dependent- Osteocalcin in the Relation Between Bone Metabolism and Metabolic Disorders. Rev Endocr Metab Disord (2020) 21:117–25. doi: 10.1007/s11154-019-09517-9 31761961

[3] Hauschka PV Lian JB Cole DE Gundberg CM. Osteocalcin and Matrix Gla Protein: Vitamin K-Dependent Proteins in Bone. Physiol Rev (1989) 69:990–1047. doi: 10.1152/physrev.1989.69.3.990 2664828

[4] Morris DP Stevens RD Wright DJ Stafford DW. Processive Post-Translational Modification. Vitamin K-Dependent Carboxylation of a Peptide Substrate. J Biol Chem (1995) 270:30491–8. doi: 10.1074/jbc.270.51.30491 8530480

[5] Massera D Biggs ML Walker MD Mukamal KJ Ix JH Djousse L. Biochemical Markers of Bone Turnover and Risk of Incident Diabetes in Older Women: The Cardiovascular Health Study. Diabetes Care (2018) 41:1901–8. doi: 10.2337/dc18-0849 PMC6105330 30002202

[6] Guo H Wang C Jiang B Ge S Cai J Zhou Y. Association of Insulin Resistance and β-Cell Function With Bone Turnover Biomarkers in Dysglycemia Patients. Front Endocrinol (Lausanne) (2021) 12:554604. doi: 10.3389/fendo.2021.554604 33841321 PMC8027237

[7] Kunutsor SK Apekey TA Laukkanen JA. Association of Serum Total Osteocalcin With Type 2 Diabetes and Intermediate Metabolic Phenotypes: Systematic Review and Meta-Analysis of Observational Evidence. Eur J Epidemiol (2015) 30:599–614. doi: 10.1007/s10654-015-0058-x 26085114

[8] Mizokami A Mukai S Gao J Kawakubo-Yasukochi T Otani T Takeuchi H. GLP-1 Signaling Is Required for Improvement of Glucose Tolerance by Osteocalcin. J Endocrinol (2020) 244:285–96. doi: 10.1530/joe-19-0288 31693486

[9] Pi M Kapoor K Ye R Nishimoto SK Smith JC Baudry J. Evidence for Osteocalcin Binding and Activation of GPRC6A in β-Cells. Endocrinology (2016) 157:1866–80. doi: 10.1210/en.2015-2010 PMC4870875 27007074

[10] Lee NK Sowa H Hinoi E Ferron M Ahn JD Confavreux C. Endocrine Regulation of Energy Metabolism by the Skeleton. Cell (2007) 130:456–69. doi: 10.1016/j.cell.2007.05.047 PMC2013746 17693256

[11] Kord-Varkaneh H Djafarian K Khorshidi M Shab-Bidar S. Association Between Serum Osteocalcin and Body Mass Index: A Systematic Review and Meta-Analysis. Endocrine (2017) 58:24–32. doi: 10.1007/s12020-017-1384-4 28822067

[12] Hiam D Landen S Jacques M Voisin S Alvarez-Romero J Byrnes E. Osteocalcin and Its Forms Respond Similarly to Exercise in Males and Females. Bone (2021) 144:115818. doi: 10.1016/j.bone.2020.115818 33338665

[13] World Health Organization. Definition, Diagnosis and Classification of Diabetes Mellitus and Its Complications. Part 1: Diagnosis and Classification of Diabetes Mellitus. Geneva, Switzerland: WHO (1999).

[14] American Diabetes Association. 2. Classification and Diagnosis of Diabetes: Standards of Medical Care in Diabetes-2018. Diabetes Care (2018) 41:S13–27. doi: 10.2337/dc18-S002 29222373

[15] Lowe WL Jr. Scholtens DM Kuang A Linder B Lawrence JM Lebenthal Y. Hyperglycemia and Adverse Pregnancy Outcome Follow-Up Study (HAPO FUS): Maternal Gestational Diabetes Mellitus and Childhood Glucose Metabolism. Diabetes Care (2019) 42:372–80. doi: 10.2337/dc18-1646 PMC6385693 30655380

[16] Lowe WL Jr Scholtens DM Lowe LP Kuang A Nodzenski M Talbot O. Association of Gestational Diabetes With Maternal Disorders of Glucose Metabolism and Childhood Adiposity. Jama (2018) 320:1005–16. doi: 10.1001/jama.2018.11628 PMC6143108 30208453

[17] Yu Y Arah OA Liew Z Cnattingius S Olsen J Sørensen HT. Maternal Diabetes During Pregnancy and Early Onset of Cardiovascular Disease in Offspring: Population Based Cohort Study With 40 Years of Follow-Up. BMJ (2019) 367:l6398. doi: 10.1136/bmj.l6398 31801789 PMC6891797

[18] Aroda VR Christophi CA Edelstein SL Zhang P Herman WH Barrett-Connor E. The Effect of Lifestyle Intervention and Metformin on Preventing or Delaying Diabetes Among Women With and Without Gestational Diabetes: The Diabetes Prevention Program Outcomes Study 10-Year Follow-Up. J Clin Endocrinol Metab (2015) 100:1646–53. doi: 10.1210/jc.2014-3761 PMC4399293 25706240

[19] Bao W Li S Chavarro JE Tobias DK Zhu Y Hu FB. Low Carbohydrate-Diet Scores and Long-Term Risk of Type 2 Diabetes Among Women With a History of Gestational Diabetes Mellitus: A Prospective Cohort Study. Diabetes Care (2016) 39:43–9. doi: 10.2337/dc15-1642 PMC4686844 26577416

[20] American Diabetes Association. 14. Management of Diabetes in Pregnancy: Standards of Medical Care in Diabetes-2021. Diabetes Care (2021) 44:S200–10. doi: 10.2337/dc21-S014 33298425

[21] Winhofer Y Handisurya A Tura A Bittighofer C Klein K Schneider B. Osteocalcin Is Related to Enhanced Insulin Secretion in Gestational Diabetes Mellitus. Diabetes Care (2010) 33:139–43. doi: 10.2337/dc09-1237 PMC2797959 19808925

[22] Srichomkwun P Houngngam N Pasatrat S Tharavanij T Wattanachanya L Khovidhunkit W. Undercarboxylated Osteocalcin Is Associated With Insulin Resistance, But Not Adiponectin, During Pregnancy. Endocrine (2016) 53:129–35. doi: 10.1007/s12020-015-0829-x 26708046

[23] Tabatabaei N Giguère Y Forest JC Rodd CJ Kremer R Weiler HA. Osteocalcin Is Higher Across Pregnancy in Caucasian Women With Gestational Diabetes Mellitus. Can J Diabetes (2014) 38:307–13. doi: 10.1016/j.jcjd.2014.02.007 24986803

[24] Metzger BE Gabbe SG Persson B Buchanan TA Catalano PA Damm P. International Association of Diabetes and Pregnancy Study Groups Recommendations on the Diagnosis and Classification of Hyperglycemia in Pregnancy. Diabetes Care (2010) 33:676–82. doi: 10.2337/dc09-1848 PMC2827530 20190296

[25] Alberti KG Zimmet PZ. Definition, Diagnosis and Classification of Diabetes Mellitus and Its Complications. Part 1: Diagnosis and Classification of Diabetes Mellitus Provisional Report of a Who Consultation. Diabetes Med (1998) 15:539–53. doi: 10.1002/(sici)1096-9136(199807)15:7<539::Aid-dia668>3.0.Co;2-s 9686693

[26] Nagasue K Inaba M Okuno S Kitatani K Imanishi Y Ishimura E. Serum N-Terminal Midfragment vs. Intact Osteocalcin Immunoradiometric Assay as Markers for Bone Turnover and Bone Loss in Hemodialysis Patients. BioMed Pharmacother (2003) 57:98–104. doi: 10.1016/s0753-3322(02)00344-x 12842495

[27] Ferron M McKee MD Levine RL Ducy P Karsenty G. Intermittent Injections of Osteocalcin Improve Glucose Metabolism and Prevent Type 2 Diabetes in Mice. Bone (2012) 50:568–75. doi: 10.1016/j.bone.2011.04.017 PMC3181267 21550430

[28] Zhou B Li H Liu J Xu L Guo Q Zang W. Autophagic Dysfunction Is Improved by Intermittent Administration of Osteocalcin in Obese Mice. Int J Obes (Lond) (2016) 40:833–43. doi: 10.1038/ijo.2016.1 26740123

[29] Yasutake Y Mizokami A Kawakubo-Yasukochi T Chishaki S Takahashi I Takeuchi H. Long-Term Oral Administration of Osteocalcin Induces Insulin Resistance in Male Mice Fed a High-Fat, High-Sucrose Diet. Am J Physiol Endocrinol Metab (2016) 310:E662–75. doi: 10.1152/ajpendo.00334.2015 26884384

[30] Sabek OM Nishimoto SK Fraga D Tejpal N Ricordi C Gaber AO. Osteocalcin Effect on Human β-Cells Mass and Function. Endocrinology (2015) 156:3137–46. doi: 10.1210/en.2015-1143 26151356

[31] Liu C Wo J Zhao Q Wang Y Wang B Zhao W. Association Between Serum Total Osteocalcin Level and Type 2 Diabetes Mellitus: A Systematic Review and Meta-Analysis. Horm Metab Res (2015) 47:813–9. doi: 10.1055/s-0035-1564134 26372899

[32] Catalano PM Tyzbir ED Roman NM Amini SB Sims EA. Longitudinal Changes in Insulin Release and Insulin Resistance in Nonobese Pregnant Women. Am J Obstet Gynecol (1991) 165:1667–72. doi: 10.1016/0002-9378(91)90012-g 1750458

[33] Agha-Jaffar R Oliver N Johnston D Robinson S. Gestational Diabetes Mellitus: Does an Effective Prevention Strategy Exist? Nat Rev Endocrinol (2016) 12:533–46. doi: 10.1038/nrendo.2016.88 27339886

[34] McIntyre HD Catalano P Zhang C Desoye G Mathiesen ER Damm P. Gestational Diabetes Mellitus. Nat Rev Dis Primers (2019) 5:47. doi: 10.1038/s41572-019-0098-8 31296866

[35] Vounzoulaki E Khunti K Abner SC Tan BK Davies MJ Gillies CL. Progression to Type 2 Diabetes in Women With a Known History of Gestational Diabetes: Systematic Review and Meta-Analysis. BMJ (2020) 369:m1361. doi: 10.1136/bmj.m1361 32404325 PMC7218708

[36] Saucedo R Rico G Vega G Basurto L Cordova L Galvan R. Osteocalcin, Under-Carboxylated Osteocalcin and Osteopontin Are Not Associated With Gestational Diabetes Mellitus But Are Inversely Associated With Leptin in Non-Diabetic Women. J Endocrinol Invest (2015) 38:519–26. doi: 10.1007/s40618-014-0220-4 25480426

[37] Liu JM Rosen CJ Ducy P Kousteni S Karsenty G. Regulation of Glucose Handling by the Skeleton: Insights From Mouse and Human Studies. Diabetes (2016) 65:3225–32. doi: 10.2337/db16-0053 PMC5860442 27959858

[38] Delmas PD Eastell R Garnero P Seibel MJ Stepan J. The Use of Biochemical Markers of Bone Turnover in Osteoporosis. Committee of Scientific Advisors of the International Osteoporosis Foundation. Osteoporos Int (2000) 11(Suppl 6):S2–17. doi: 10.1007/s001980070002 11193237

