# Supplementary material for: Consistently Low Levels of Osteocalcin From Late Pregnancy to Postpartum Are Related to Postpartum Abnormal Glucose Metabolism in GDM Patients
Source: Front Endocrinol (Lausanne). 2022 Mar 7;13:803624. doi: 10.3389/fendo.2022.803624 (PMC8936089; doi:10.3389/fendo.2022.803624)
Supplement: Supplementary file 1 [file Table_1.docx]

**Supplementary Table 1.** Linear mixed model of risk factors for longitudinal changes in serum OC concentrations.

|  | β | 95% CI | | P value |
| --- | --- | --- | --- | --- |
| Intercept | 21.42 | 13.57 | 29.26 | <0.001 |
| Primiparity | 2.09 | 0.96 | 3.22 | <0.001 |
| (reference category multiparity) |  |  |  |  |
| No family history of diabetes | -0.23 | -1.49 | 1.04 | 0.726 |
| (reference category family history of diabetes) |  |  |  |  |
| Postpartum AGM | -1.70 | -2.78 | -0.62 | 0.002 |
| (reference category postpartum NGT) |  |  |  |  |
| Age (years) | -0.19 | -0.32 | -0.06 | 0.005 |
| Time | -9.9 | -10.44 | -9.35 | <0.001 |
| (reference postpartum) |  |  |  |  |
| Pre-BMI (kg/m^2^) | 0.08 | -0.08 | 0.23 | 0.331 |
| HbA1c in late pregnancy (%) | 1.36 | 0.23 | 2.5 | 0.019 |

AGM, abnormal glucose metabolism; NGT, normal glucose tolerance; Pre-BMI, BMI before pregnancy.

β coefficient for “AGM” represents the difference of OC levels between AGM and NGT controls at postpartum;

β coefficient for “Time” represents the longitudinal change of OC levels from later pregnancy to postpartum among NGT controls;

There was no significant interaction between Group (AGM or NGT) and Time, the interaction therefore was not included in the final model.
